# A holistic approach to supporting death in the home: Implementation of a home hospice program in a northern community–A study protocol

**DOI:** 10.1371/journal.pone.0306553

**Published:** 2024-10-29

**Authors:** Shannon Freeman, Donna Flood, Davina Banner, Aderonke Agboji, Erin Anderlini, Mathew Sargent, Leah Chambers, Anhelina Maksymova, Emma Rossnagel

**Affiliations:** 1 School of Nursing, University of Northern British Columbia, Prince George, BC, Canada; 2 Prince George Hospice Palliative Care Society, Prince George, BC, Canada; 3 Centre for Technology Adoption for Aging in the North, University of Northern British Columbia, Prince George, BC, Canada; BP Koirala Memorial Cancer Hospital, NEPAL

## Abstract

**Introduction:**

To enhance support for individuals at end of life to die at home, a new community-driven home-based hospice program was developed–Home Hospice. This wholistic hospice care program, co-designed by hospice care staff, community end-of-life care providers, researchers, and health systems decision-makers, will provide around the clock care to clients and their families.

**Methods and analysis:**

This mixed methods study, guided by a process evaluation framework, will use secondary client and caregiver data collected as part of regular Home Hospice program processes, as well as primary interview data collected from caregivers at least three months post-death of the client and from hospice staff and volunteers involved in the Home Hospice program. Analysis will include descriptive statistical analysis of quantitative data and thematic analysis employing an inductive and a deductive qualitative analysis approach.

**Ethics:**

Ethics approval has been received from the University of Northern British Columbia (File No: 6009117). All individuals and caregivers who enroll in the Home Hospice program will be invited to participate in this study. At three months post-death of the client, hospice staff will contact caregivers to ask if they would like to participate in a one-on-one interview with a research team member. Staff and volunteers will also be eligible to participate in one-on-one interviews with research team members. De-identified participant data will be provided by hospice staff to the research team. All participants will provide informed consent prior to participation in the study.

**Dissemination:**

This study will use an integrated Knowledge Translation/Knowledge Mobilization (iKTKM) lens wherein the research team will work closely and meaningfully with knowledge users and community partners at all stages. Evidence-based and targeted iKTKM products will be created, including community event presentations and social media posts to share findings with participant and community audiences. Further, findings will be disseminated at conferences, workshops, and policy rounds with academic and decision-making audiences. Preliminary and full project findings will be published in high-quality open access journals.

**Strengths and limitations of this study:**

- Co-design of Home Hospice program and evaluation metrics involves researchers, hospice staff, health systems decision-makers, and community members.
- Evaluation metrics involve prospective collection of service utilization from client, family, hospice, and health systems perspectives.
- Integrated knowledge translation approach which frames the knowledge dissemination plan for the study findings focuses on a range of audiences, including local citizens, hospice and community end-of-life care providers, health systems decision-makers, and academic professionals.
- As a one-year pilot study, the number of clients who may be able to participate in this program will be approximately 50 persons.

## Introduction

Hospice palliative care is “the combination of active and compassionate therapies intended to comfort and support persons and families who are living with, or dying from, a progressive life-limiting illness, or are bereaved” [1]. This includes “whole-person health care that aims to relieve suffering and improve the quality of living and dying” [2]. Underpinned by a wholistic approach, the provision of hospice palliative care not only supports high-quality pain and symptom management but also supports individuals’ physical, psycho-social, and spiritual needs as they prepare for and navigate their life-limiting illness and dying journey. Providing high-quality hospice palliative care also supports the family and friends of the individual receiving palliative care as they cope with grief and loss during the individual’s illness and bereavement [3].

While most individuals express a desire to die at home or in a home-like setting, a substantial number of individuals continue to die in acute care settings [4]. In Canada, although the first interprofessional palliative care services were offered in 1974 [2], access to hospice palliative care remains fragmented and is not accessible to all individuals, especially those who wish to die at home. Of those who are able to access palliative care services in Canada, nearly two thirds received palliative care in an acute care setting in their last month of life [5]. CIHI (2018) reports that fewer than one in six people (15%) received publicly funded home care during their last year of life. This is in great contrast to findings from the United States, where Bhatnagar and Lagnese noted that over 50% of patients who received hospice care were cared for in their own home [5]. Receipt of palliative care [6] has been shown to lead to great financial savings and reductions of acute care health resource expenditures. This includes reductions in the overall length of stay in an acute care setting, the number of ICU admissions, unnecessary diagnostic tests, and inappropriate disease-focused interventions [7]. Hospice palliative care and supports should not be limited to care in the acute care setting, but instead, be made available to support the individual in the setting of their choice.

In rural and northern communities across Canada, there is an even greater gap in the availability of hospice care and support for those at end of life. The absence of equitable access to in-hospice care is amplified by the lack of community services and supports intended to equip those who wish to, die at home. It is well established that this shortfall of resources can negatively impact quality of care and well-being at end of life and increase the number of individuals [7] who may die on their way to the hospital, in the emergency department, or in acute care settings. To support high-quality end-of-life care, persons who are in the terminal stage of their illness and who wish to die at home may require an increasing level of hospice services and supports. Informal family and friend caregivers, who play an integral role in supporting persons to die at home, also require hospice supports, including access to information, education, resources, emotional support, and assistance in providing care.

Therefore, in partnership with leaders from an independent hospice society, an interdisciplinary team co-created an in-home hospice support program in a northern geographically isolated medium-sized city in Western Canada. This partnership includes bereaved family caregivers, community members and volunteers, hospice clinicians and staff, health systems decision-makers, physicians, and academics, who collectively sought to enhance accessibility to hospice care in the community through the provision of person and family-centred psychosocial care, 24-hour crisis support, and enhanced grief and wellness supports in the client’s home. It was determined that this program will offer access to pain and symptom experts, as well as provide access to health care and grief support professionals 24 hours a day, 7 days a week, to better support clients who desire to die in their own home, and their families. The goals of the newly designed “Home Hospice” program include:

- To allow individuals the resources and supports to die comfortably at home if that is the place of their choosing;
- To provide high-quality pain and symptom management and 24-hour crisis support in the individual’s home;
- To educate and support families thereby reducing anxiety and fear about the dying process and preventing/reducing risk of complicated grief; and
- To decrease unplanned visits to the emergency department and death in the acute care setting at end of life.

Following, the objectives of this study will be to evaluate the:

- Feasibility, useability, and functionality of the Home Hospice program to support death in the home;
- Ability of the Home Hospice program to support more palliative patients to die at home;
- Level of support needed for caregivers to address their emotional, educational, and clinical support needs on a 24 hour/7 day a week basis; and
- Impact on number emergency department visits and acute care admissions, after implementation of the Home Hospice program.

## Methods and analysis

### Study design

This mixed methods study is guided by Moore and colleagues’ process evaluation framework for planning, designing, conducting, analysing, and reporting findings from the first-year pilot of the Home Hospice program [8]. This framework was selected as it emphasizes the relationships between implementation, mechanisms, and context as the key components of the evaluation [8]. We will articulate the functioning of the program and identify areas of innovation in hospice care service delivery in the home. The guiding study questions organized by the three components of Moore’s framework will be:

1. Implementation–To describe the implementation process of the Home Hospice program.
2. Mechanisms of impact–To identify how the delivered Home Hospice program produces change at the individual, community, and health systems levels.
3. Context–To assess how the northern context affects implementation and client, caregiver, and health systems outcomes.

### Sample recruitment

In the first year of this pilot, up to 10 persons may be enrolled in the Home Hospice program at any one time. The maximum number of 10 participants simultaneously enrolled in the program was determined ensure that there is adequate staff available for planned and unplanned visits. This includes 24 hours a day on call nursing staff available to respond to unscheduled needs as required. It is projected that approximately 50 individuals will participate in the Home Hospice program in the first year. We will include secondary data collected from all clients and caregivers who consent to participate in the study. Further, all bereaved caregivers will be invited to participate in one interview at least three months following the client’s death. The actual number of participants may increase or decrease as the program expands. We will also engage 10–12 hospice staff and/or volunteers involved in design and/or program delivery of the Home Hospice program. Additionally, any other individual involved with the co-development, or delivery, of the Home Hospice program will be eligible for participation in one-on-one interviews.

#### Client and caregiver recruitment

As part of the admission to the Home Hospice program, all clients and caregivers will be invited to participate in research and evaluation activities. Data from consented clients and caregivers will be collected from a variety of sources during the client’s participation in the Home Hospice program. All data will be de-identified by hospice staff and included as secondary data in this study.

As part of regular hospice practice, hospice staff will check in with caregivers approximately three months following the death of the client. At this time, caregivers who indicated willingness to participate in research activities during program admission will be subsequently invited to participate in a one-on-one interview with research staff. Should a caregiver indicate interest, the hospice staff will share their name and preferred method of contact (e-mail or phone) with research staff. A member of the research team will contact interested caregivers to describe the project, share an information letter, and request their consent to participate in a one-on-one interview. Once written informed consent has been received, the interview will be scheduled with the caregiver participant, based on their preferences (in-person at the hospice house or by Zoom). Interviews are expected to last from 30 minutes to one hour in duration.

#### Staff and volunteer recruitment

Staff and volunteers involved in providing care to participants and/or family (e.g. nursing staff, volunteer visitors) and/or who are connected to the Home Hospice program (e.g. management or program support staff) will be invited to participate. All staff and volunteers with knowledge or experience related specifically to the Home Hospice program will be eligible to participate. A poster will be shared with staff and volunteers, and announcements about the research study will be disseminated through e-mail and at staff meetings. Consenting participant staff and volunteers will be asked to complete an interview with the research team to discuss their experience with the Home Hospice program. Interview questions will centre around their experience with the Home Hospice program and will be non-invasive in nature. Further, any other individuals involved in the co-design of the Home Hospice program will also be invited via e-mail and word of mouth to participate in the study.

#### Participant compensation

All participants who complete the one-on-one interview will be eligible to receive a $35 e-gift card as recognition of their time and the resources required to participate (e.g. internet connectivity for virtual interview, gas and/or bus fare to attend in-person).

### Sources of data

We will collect secondary de-identified client and caregiver data. Sources for this data will include client referral and intake forms, daily client reports, Home Hospice program monthly reports, closed file reviews, aggregate caregiver post-bereavement survey results, and volunteer journal entries. Further, we will seek to access health systems level aggregate reports from our regional health authority regarding the number of palliative hospital deaths and ER visits. The program is in a geographically isolated city where the majority of deaths currently occur at the University hospital, the largest acute care centre in an area of 600,000km^2^ which serves a population of 300,000 individuals. Examples of data to be collected from the various secondary data sources are described in Table 1.

**Table 1:** Secondary data sources and anticipated data collection points.

Secondary Data Source	Data to be collected
Caregiver survey	• Level of caregiver distress • Comfort with program • Experiences seeking support in a crisis • Use of care and services outside Home Hospice program
Volunteer Journal	• Descriptions of caregiver burden • Duties provided to support client and caregiver
Daily and Monthly Staff Report	Reasons for planned and unplanned visits • Use of in-home technologies • Documented daily calls • Any delays in receiving care • Timeliness of response for symptom management • Services and supports requested by client and caregiver • Number of clients enrolled in the program • Number of clients on the waitlist
Medical Chart and Closed File Review	• Location of care prior to referral to Home Hospice program • Reason for client discharge from program (e.g. death, client withdrew, transition to other site for care) • Duration client enrolled in program • Place of death • Reason for referral • Reason for waitlist • Ambulance, emergency department, and acute care utilization

Further, qualitative data will be collected from bereaved caregivers at a minimum of three months following the death of the client, and from hospice staff and volunteers engaged in the delivery or design of the Home Hospice program. Interviews will examine motivations for participating in the Home Hospice program, benefits and challenges experienced during the program, and impact of receiving support at home on client care as well as client and caregiver well-being. Interviews will also capture caregiver perspectives on communication with staff and use of communication technologies to enhance care. Finally, opportunity will be provided to discuss suggestions for program adjustments and improvements. Copies of the semi-structured interview script are available at request from the corresponding author.

### Data analysis

#### Quantitative analysis

Secondary data, organized using Microsoft Excel [9] and SPSS software [10], will be summarized using descriptive statistics. Due to the anticipated small sample size of participants in the study, the use of inferential statistics is not expected. However, should sample size allow, inferential statistics will be conducted where possible. Tables and charts will be used to visually summarize findings. Where sample sizes allow, findings will be stratified by client sex or gender.

#### Qualitative analysis

Interviews will be transcribed and imported into NVivo coding software [11] to organize the data and support analysis. Data will be coded and analyzed thematically using Braun and Clarke’s six steps [11, 12]. Familiarization with the data will involve close reading and re-reading of the data to inform the generation of an initial code book. To establish intercoder reliability, limit bias, and establish rigour, two researcher team members will analyze the same five transcripts and code them separately to begin an inductive-deductive analysis process [13]. This analysis process will incorporate both a pre-determined (deductive) strategy that will draw from Moore et al.’s theoretical framework to inform interpretation of the data and will also employ an emergent (inductive) strategy that will identify representative data to support findings and deepen understanding of themes. The researchers will discuss commonalities and differences between their preliminary findings, come to an agreement on the names of themes, and develop a single coding framework. This coding framework will then be applied to all interview transcripts. A thematic map will be developed. Themes will be reviewed by the larger research team in an ongoing and iterative manner to generate clear descriptions and defining of the themes.

Data from both the quantitative and qualitative research activities will be integrated to create a comprehensive analysis of the home hospice program. We will conduct concurrent data collection and analysis, seeking to integrate insights from each to garner a more fulsome understanding of experiences, as well as leveraging these insights to inform ongoing data collection and analysis. For example, sex and gender-based analysis will be conducted if participant numbers allow. Integration of these insight may identify opportunities for future inquiry, including further recruitment of participants with specific characteristics or experiences. We recognize the limitation of this study design to the analysis plan given the lack of inclusion of a control group at this time.

### Data management plan

All efforts to ensure the security of participants’ information will be taken, including password-protected meeting invites and safeguarding the recordings of the Zoom (audio and video) interviews by storing them on a password-protected server. All study data, including interview audio recordings and transcripts, will be stored on a secure server maintained by the University Information Technology Services. The de-identified data will be provided to the research team directly from hospice staff through a secure file transfer using Sync. Access to all data will be restricted to authorized study team members.

## Knowledge dissemination

We will use an integrated Knowledge Translation/ Knowledge Mobilization (iKTKM) lens wherein we work closely and meaningfully at all stages with our knowledge user and community partners. We will share project-related information through our intersectoral networks to leverage the study’s findings to inform program delivery and practice change within the health system. Where possible, we will delineate findings to describe how sex and gender, in addition to other key demographic characteristics, are impacted by the Home Hospice program. We will generate evidence-based and targeted iKTKM products, including presenting findings at community events and through social media to target participant and community audiences. Further, we will disseminate findings at conferences, workshops, and policy rounds with decision and policy-maker audiences, as well as a publish peer-reviewed articles in high-quality open access journals.

## Supporting information

S1 Checklist Human participants research checklist. (DOCX)
